# Identifying gene expression-based biomarkers in online learning environments

**DOI:** 10.1093/bioadv/vbac074

**Published:** 2022-10-13

**Authors:** Luca Cattelani, Vittorio Fortino

**Affiliations:** Institute of Biomedicine, School of Medicine, University of Eastern Finland, Kuopio, Finland; Institute of Biomedicine, School of Medicine, University of Eastern Finland, Kuopio, Finland

## Abstract

**Motivation:**

Gene expression-based classifiers are often developed using historical data by training a model on a small set of patients and a large set of features. Models trained in such a way can be afterwards applied for predicting the output for new unseen patient data. However, very often the accuracy of these models starts to decrease as soon as new data is fed into the trained model. This problem, known as concept drift, complicates the task of learning efficient biomarkers from data and requires special approaches, different from commonly used data mining techniques.

**Results:**

Here, we propose an online ensemble learning method to continually validate and adjust gene expression-based biomarker panels over increasing volume of data. We also propose a computational solution to the problem of feature drift where gene expression signatures used to train the classifier become less relevant over time. A benchmark study was conducted to classify the breast tumors into known subtypes by using a large-scale transcriptomic dataset (∼3500 patients), which was obtained by combining two datasets: SCAN-B and TCGA-BRCA. Remarkably, the proposed strategy improves the classification performances of gold-standard biomarker panels (e.g. PAM50, OncotypeDX and Endopredict) by adding features that are clinically relevant. Moreover, test results show that newly discovered biomarker models can retain a high classification accuracy rate when changing the source generating the gene expression profiles.

**Availability and implementation:**

github.com/UEFBiomedicalInformaticsLab/OnlineLearningBD.

**Supplementary information:**

Supplementary data are available at *Bioinformatics Advances* online.

## 1 Introduction

Omics technologies have already shown a great potential for application across diverse areas of human health, including disease diagnosis, prognosis and therapeutic selection. In particular, RNA-based technologies, which allow precise measurements for millions of within-cell transcriptomics features, such as genes, miRNA, etc., can be utilized to feed machine learning (ML)-based solutions, which in turn can add robustness (value) to the conventional classification of cancer subtypes (Cascianelli *et al.*, 2020; Jiao *et al.*, 2020). Gene expression-based biomarkers exhibiting higher accuracy in cancer diagnosis are often discovered by using ML methods. However, the adopted ML paradigm learns in isolation, by constraining the discovery and training phases to a single dataset with small sample sizes. It also assumes that selected biomarkers and corresponding ML models do not change over time (Liu, 2017). However, very often the initial training set can be further extended by using external data sources. Moreover, new RNA-seq profiles can be generated for testing purposes, to provide RNA-seq-based clinical diagnostics. Newly generated (or discovered) data should then be exploited for model adaptation. In this case, it is important to deploy ML-based biomarker models that can continuously learn and evolve based on the input of increasing amounts of data, while retaining previously learned knowledge.

From a biological perspective, using biomarker models with capacity for adaptation is extremely relevant. Many chronic diseases, such as cancer, are not just one disease, but many; and despite advances in research, we still need to learn a lot about their complexity. Moreover, these diseases may evolve over time (e.g. environmental factors, new habits and diets, etc.), requiring a continuous adaption of previously learned biomarker panels. Furthermore, the limited set size of existing datasets (e.g. TCGA-BRCA) may not be sufficient to explain this complexity and, consequently, the biomarkers we presently consider important may perform poorly on newly generated data. A simple solution to these technical and scientific gaps is to repeat the whole discovery and training process every time a new batch of data is available. Such a solution (or practice) increases the chance of prolonging indefinitely the process of translating biomarkers to clinical practice. Moreover, it does not comply with the regulatory Framework for Modifications to Artificial Intelligence/Machine Learning (AI/ML)-Based Software as a Medical Device (SaMD). ML-based biomarker models may be classified as (AI/ML)-Based SaMD (https://www.fda.gov/medical-devices/software-medical-device-samd/artificial-intelligence-and-machine-learning-software-medical-device). An important feature SaMD should have, is the ability to adapt to changes, to account for post-released modifications, avoiding the need for a new clearance or approval. A solution to model adaption in ML-driven biomarker discovery, is the continual learning (CL) paradigm. CL is the ability of a model to autonomously learn and adapt as new data (for testing purposes) arrive. Most importantly, the CL paradigm allows to conduct in parallel model validation and adaptation, and therefore could allow an early utilization of ML-based biomarker models, facilitating and increasing their acceptance.

Here, we propose a computational framework to address the problem of feature drift detection in ML-driven biomarker discovery. Feature drifts occur whenever a subset of features, in these case RNA-seq-based biomarkers, becomes or ceases to be, relevant to the concept to be learned. These events are addressed by using an online feature selection system enabling the training of models that ignores irrelevant features and account for the newly relevant ones. We tested our method on large-scale transcriptomic datasets for the classification of breast cancer patients into five well-known subtypes, namely luminal A, luminal B, HER-2, basal and normal breast like. The selected datasets are based on two population-based cohort studies: SCAN-B (Brueffer *et al.*, 2018) and TCGA-BRCA. Both studies provide RNA-seq profiles of breast cancer tissues. In total, they amount to more than 4000 gene expression profiles.

## 2 Methods

### 2.1 Data collection and preparation

Two large-scale gene expression datasets characterizing tumor tissues of breast cancer patients were used to implement the proposed case study. The first dataset was retrieved from the Gene Expression Omnibus database (GSE96058). It includes gene expression profiles of more than 3k breast cancer patients characterized by the Sweden Cancerome Analysis Network—Breast (SCAN-B) consortium, a multicenter prospective study with longsighted aims to analyze breast cancers with next-generation genomic technologies for translational research in a population-based manner and integrated with healthcare. We downloaded the dataset available as FPKM-log2-transformed data. The second dataset includes normalized TCGA RNA-Seq data of breast cancer patients (BRCA) downloaded with the R-package TCGAbiolinks (Mounir *et al.*, 2019). The retrieved data consisted of FPKM values, which were then log-transformed and standardized before their use. Finally, we transformed both datasets to become *Z*-scores, to facilitate the ML-base evaluation process. Furthermore, a common set of 17 345 genes was selected to make sure that both datasets (SCAN-B and TCGA-BRCA) are based on the same set of features. The class label indicating the breast cancer subtype for each omic profile was defined based on The 2013 St Gallen classification of intrinsic subtypes, which consider the status of the ER and PgR receptors, HER2 and Ki-67 (Yersal and Barutca, 2014). These clinical markers were available for both datasets and included in the corresponding phenotype file. However, for some omic profile the information of these tumor markers is missing or the combination of their status cannot be linked with one of the four subtypes: Luminal A, Luminal B, Basal-like and Her2-positive. These unlabeled profiles were discarded. The two gene expression datasets were finally concatenated, obtaining 3356 patient-based gene expression profiles (SCAN-B: 2822; TCGA: 534).

### 2.2 The proposed FDD-ES algorithm

The proposed dynamic feature selection system consists of two components: (i) a feature drift detector (FDD) and (ii) an ensemble learning (ES) method, which uses online learning algorithms to build and train base classifiers. The FDD is implemented upon the Landmark-based Feature Drift Detector (LFDD) method (Barddal *et al.*, 2015), which aims to split a stream into chunks and perform feature selection within those chunks, in order to determine the most discriminative subset of features of a stream, and train a new classifier exclusively with them. The FDD receives as input a data stream *S*, define landmark window size *W*, an heuristic goodness function *Q(·)*, which implements, in our extended version of the LFDD method, the Minimum Redundancy and Maximum Relevance (mRMR) feature selection method (Ding and Peng, 2003). During the training step, instances (*x_i_, y_i_*) retrieved from a data stream are stored in an instance buffer and used for feature selection and classification. The FDD takes in input the information about a known biomarker model (Pam50). Then, a classification event is triggered if newly selected features include genes that are not included in known gene signatures (Pam50) or that were not included in feature set computed in previous data chunks. In order to prevent catastrophic forgetting (Kirkpatrick *et al.*, 2017), newly discovered feature sets (or biomarker models) are used to train new classifiers that will be then added to an ES system (Krawczyk and Cano, 2018). In our case study, we model a classifier, namely *C*_KB_, which is implemented by combining an online learning algorithm with the gene signature Pam50. Then, we train an RF-based model for each newly discovered feature set, namely *C_F_*. Finally, a soft voting schema is applied to merge the class probabilities generated by each classifier (*C*_KB_, *C_F_*_1_, *C_F_*_2_, *C_F_*_3_, etc.). The output class will be the prediction based on the average of probability given to that class. Each RF models is trained with 500 trees and *max-feature* equal to the square root of total number of features in our dataset. The FDD-ES relies on two main hyper-parameters: the window size *W* and the number *R* of previously trained classifiers to be used for the prediction step. The FDD-ES algorithm was implemented in python by using two ML modules: *sklearn* (Pedregosa *et al.*, 2011) and *river* (https://riverml.xyz/).

### 2.3 The adopted online learning algorithm

The proposed FDD-ES approach was tested in combination with a popular online supervised learning algorithm, which combines the Hoeffding Tree classifier with ADaptive WINdowing (ADWIN). The Very Fast Decision Tree (VFDT) a.k.a. the Hoeffding Tree classifier (Domingos and Hulten, 2000) is the core part of many online learning algorithms. It starts with a decision tree with only the root node and grows it incrementally as the samples are processed.

A leaf node is split on a feature if the probability of improving the predictions is equal to at least a user defined *P* with respect to not splitting or to splitting on another feature. The Hoeffding bound is used to check when this constraint is satisfied. VFDT assumes a distribution of the data that does not change over time and thus is not designed to handle feature drifting. Many approaches have been conceived to handle drifts. The Hoeffding Adaptive Tree (HAT) (Bifet and Gavaldà, 2009) is built upon the VFDT. At each node it associates estimators *A_i, j, k_* approximating the probability that a sample passing through that node has value *j* for the feature *i*, and class label *k*. These estimators are used to choose when and how to split a node, similarly to VFDT. In addition, each estimator signals when it detects a change in the distribution, and this is used to grow alternative branches. The alternative branches are monitored and substitute the original branches if they became more accurate. The estimator concept encapsulates the subproblem of change detection of a distribution together with the estimation of a significant statistics like the expected value. Many algorithms aiming to solve this problem are known. We used the ADWIN algorithm (Bifet and Gavaldà, 2007), that is based on a sample window and has the appreciable properties of automatically adapting the window size, and of compressing the information to O(log(*W*)) memory, where *W* is the current window size. We tested the HWT-ADWIN with a number of samples a leaf should observe between split attempts of 100 and a required split confidence of 0.99999 with information gain as split criterion. Bayes classifiers are used in the leaves that have seen at least 10 samples. Bayes classifiers can learn and evolve in an online manner, and they are often used as a component in the leaves of more complex tree-based models.

### 2.4 Training and validation framework

The assessment framework was implemented by using the python module river (https://riverml.xyz/). In an online learning framework, the general objective is to measure the model’s ability to generalize during the training process. This is achieved by quantifying the model’s capacity to generalize on a new observation, before updating the model with this new observation. This process is called progressive-validation (Blum *et al.*, 1999) and it is implemented in the river. The balanced accuracy was used as main evaluation metric, since we acknowledge that the classification problem of grouping breast cancer subtypes is unbalanced.

### 2.5 Parameter setting

In the proposed system, there are two major set of hyper-parameters: hyper-parameters of the online learning algorithms and hyper-parameters of the FDD-ES system. For the FDD-ES, we fixed the number of features to be selected in each chunk of data. The top 10 features generated by the mRMR algorithms were selected for each feature selection step. Then, we evaluated the performances of the proposed FDD-ES by tuning the size of a data chunk [*w*=(‘200’,‘500’)] and the number of previously trained models to be used for the prediction step [*r*=(1, 2, 3)].

### 2.6 Statistical analysis

Cox-regression analysis (Cox, 1972) was applied to verify whether the expression signature of the genes selected by the FDD-ES was associated with the overall survival of breast cancer patients in the SCAN-B cohort. This analysis was implemented by using the python module lifelines (Davidson-Pilon, 2019). Genes with the absolute value of the estimated coefficient >0.2 and *P*-value <0.01 were selected in order to build a disease risk score (He and Zuo, 2019). The risk score was constructed as follows: $\mathrm{isk}\;\mathrm{score}=\sum_{i=1}^{n}\exp_{i}\times \beta_{i}$; where *n* was the number of prognostic genes, exp_*i*_ the expression value of gene *i*, and *β_i_* the regression coefficient of gene *i* in the univariate Cox-regression analysis. A cutoff value was applied to divide the patients into groups (high- and low-risk groups), and the Kaplan–Meier method (Kaplan and Meier, 1958) was used to assess the differences in survival time of low- and high-risk breast cancer patients, and the log-rank test was used to determine the statistical significance of observed differences between the two groups.

## 3 Results

### 3.1 Online ES for biomarker discovery

**Fig. 1.**
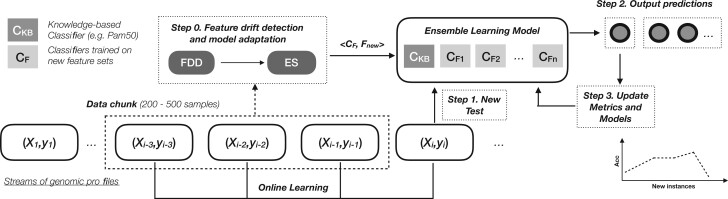
Workflow of the proposed method for dynamic feature selection in a continuous flow of RNA-seq data generated by various data sources

In this study, we propose an adaptive feature selection system for evolving biomarkers to classify patients based on a stream of gene expression profiles. The proposed approach, which is graphically illustrated in Figure 1, consists of a FDD and an online ES. The main objective is to show that online learning can constantly improve a pre-defined biomarker model, such as Pam50, or it can serve as full de novo biomarker detector. Transcriptomic streams are first divided into chunks and then the mRMR algorithm is applied to select 10 informative genes. Then, the FDD verifies whether these genes were not selected with previous chunks of data. The biomarker discovery process begins with a known biomarker model or an empty model. Newly detected features trigger a drift event and, consequently, new base classifiers are systematically trained by using these features. Base classifiers are generated by using HT-ADWIN method and then added to the online ES system. It should be noted that all models are updated after testing them on new, unseen samples. Therefore, the prediction task is carried out before updating the balanced accuracy. When predicting the cancer type of a new, unseen patient, the online classifier, which is based on a known biomarker model (e.g. Pam 50), is used in combination with the best *k* models, which were trained with newly discovered features or biomarkers. The best models are selected based on their balanced accuracy that was calculated before updating the model with the new instance. The proposed FDD-ES system prevents catastrophic forgetting in feature drifting, by making sure that previously learned biomarkers (or genes) are still in use in previously trained online models. The final biomarker model will be defined as the union between known biomarkers and those discovered during the online learning process.

### 3.2 Performances obtained with gold-standard biomarkers

**Fig. 2.**
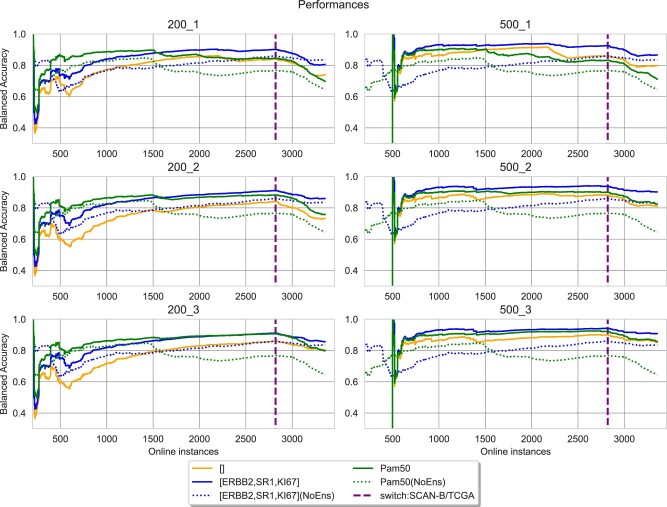
Classification performances obtained by starting FDD-ES with two basic biomarker models and an empty biomarker set. The *y*-axis reports the balanced accuracy computed as new instances are received, varying the size of a data chunk (200/500) and the number of base models used for prediction (from one to three models). The two numbers above the graphs are respectively the size of chunks and the number of best models. The accuracies of the FDD models are in solid lines while the lines for their non-ensemble counterparts are in the same color but dotted. The dashed line on the online instances indicates the switch to TCGA-BRCA data

**Fig. 3.**
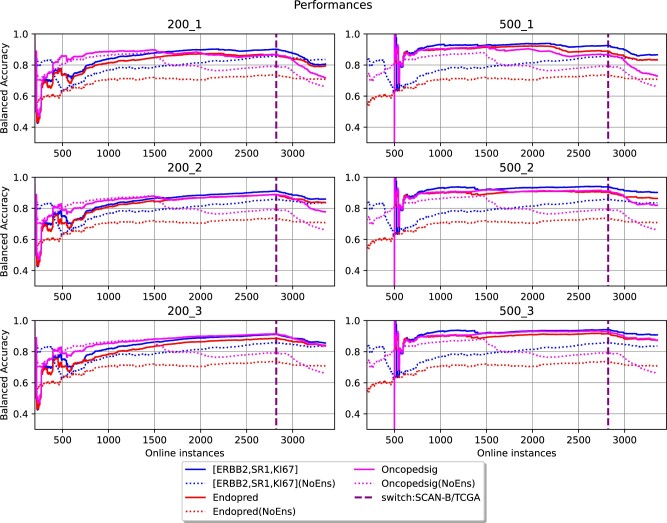
Classification performances obtained by starting FDD-ES with four basic biomarker models. The *y*-axis reports the balanced accuracy computed as new instances are received, varying the size of a data chunk (200/500) and the number of base models used for prediction (from 1 to 3 models). The two numbers above the graphs are respectively the size of chunks and the number of best models. The accuracies of the FDD models are in solid lines while the lines for their non-ensemble counterparts are in the same color but dotted. The dashed line on the online instances indicates the switch to TCGA-BRCA data

The FDD-ES was applied to learn informative biomarkers predictive of breast cancer subtypes. The breast cancer subtype were defined based on the status of the ER and PgR receptors, HER2 status and Ki-67 (Goldhirsch *et al.*, 2013), as suggested by the latest 2013 St. Gallen International Breast Cancer Conference. FDD-ES was tested with four gold-standard biomarkers, which are (ESR1, HER2, Ki-67) (here indicated as M3), PAM50 (Parker *et al.*, 2009), Endopredict (Filipits *et al.*, 2011) and OncotypeDX (Paik *et al.*, 2004). FDD-ESS was also tested without using an initial set of known biomarkers, thus serving as a full *de novo* biomarker detector. For each gold standard, the proposed online ensemble (ES) learning method was tested on two concatenated datasets [(SCAN-B, TCGA-BRCA) and (TCGA-BRCA, SCAN-B)] in a streaming fashion, to partly simulate a technological shift. The ES builds a new HAT-based classifier for each feature drift event. An FDD event was triggered each time the mRMR method detected newly gene-based biomarkers over a data stream of RNA-seq-based transcriptomic profiles. Figures 2 and 3 show the comparisons of test accuracy rates, which are computed on new unseen data. In more detail, the online learning approach that does not use FDD-ES (‘NoEns’) and the FDD-ES, which includes newly discovered biomarker models, were systematically compared.

Figure 2 includes the results obtained by using the M3, Pam50 or an empty model, while the comparison between M3 and endoPredict or OncotypeDX is reported in Figure 3. Remarkably, the FDD-ES approach systematically outperforms the online learning models that are based on gold-standard biomarker panels. This suggests that FDD-ES may enable the discovery of novel informative genes that can improve the overall test accuracy. When the switch to TCGA-BRCA samples occurs (dashed line), it is possible to observe a decrease in accuracy, which is especially evident on the model trained with Pam50. However, the online ES strategy can still retain a high classification accuracy rate. Pam50, which is a well-known biomarker panels for the classification of breast cancer subtypes, exhibits a classification accuracy that tends to decrease over time, even before the switch to TCGA-BRCA samples. This can suggest that the biomarker composition of Pam50 might not be optimal. However, the FDD-ES method can still improve the model’s performance of Pam50, by adding new informative genes.

Moreover, we observe that the model based on the features (ESR1, HER2, Ki-67) can constantly improve by analyzing new data. On the contrary, even the Pam50-based model is characterized by an accuracy rate that decreases over time. This may suggest that Pam50 includes already too many features, which may lead to overfitting issues. This might indicate that a CL process applied to biomarker discovery should probably start with a very specific and small set of biomarkers. Notably, the classification accuracy achieved by the FDD-ES with an empty model (*de novo* biomarker discovery) is also high. Although, if the window size =200, it then needs to observe at least 1000 samples before achieving a good performance. Supplementary Figures S1 and S2 include the results obtained when considering the switch from TCGA-BRCA to SCAN-B data. These results confirm that model’s performance of Pam50 decrease when changing the data source and that alternative features (or genes) are needed to improve the classification performance over SCAN-B data. Notably, FDD-ES selected biomarker panels achieve comparable accuracy to those discovered with the test ‘SCAN-B/TCGA’.

As expected, a larger window (e.g. *w* = 500), allows the identification of more informative biomarkers, since ES-based models (represented by solid lines) can achieve high accuracy rates already with the first 1000 instances. The number of the best models, which are used for the prediction step, is also important. It can help overcome the decrease in accuracy (concept drift) occurring when new unseen instances arrive from a different dataset/cohort, in this case represented by the TCGA-BRCA dataset. Indeed, when the top 2/3 models are used to make predictions, the accuracy achieved by the ES is still high over the TCGA data. It is also interesting to observe that the OncotypeDX panel starts with a high classification accuracy, but then the biomarker model’s performance starts to decrease around 1500 instances. However, the performances are improved by the ES, suggesting that key features for the classification of breast cancer subtypes are missing from the OncotypeDX signature. Remarkably, the FDD-ES can greatly improve the performances of the online model trained with the endoPredict signature.

Table 1 reports the sizes of biomarker sets that are learned with the online learning process. It also includes the number of biomarkers that are shared with the gold-standard Pam50. Overall, the size of the biomarker sets tends to increase. On the other hand, the number of newly added features tends to decrease over time. It is interesting to observe that the biomarker model, which is learned from M3 achieve better performances than those obtained by evolving the Pam50 model, indicating that probably many features of the Pam50 are correlated, and therefore smaller sets of biomarkers can achieve similar prediction accuracies (Fortino *et al.*, 2020).

**Table 1.** Size of biomarker models and their intersection with known breast cancer biomarkers

Initial model	Size (IM)	Win	Final model	Intersection with known models			
				*M3*	*Pam50*	*Endopred*	*Oncoped*
M3	3	200	44	3	3	3	3
		500	36	3	3	3	3
Pam50	50	200	85	6	50	8	14
		500	77	6	50	8	14
Endopred	12	200	55	0	2	12	1
		500	47	0	2	12	1
Oncoped	21	200	61	3	11	4	21
		500	53	3	11	4	21

### 3.3 Evaluation of selected biomarkers

**Fig. 4.**
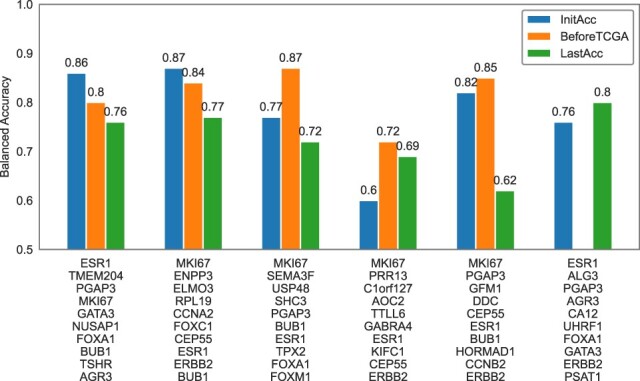
Classification performances obtained by feature sets that are learned over the stream of gene expression-based profiles. The stream of gene expression profiles is generated from two sources: SCAN-B and TCGA-BRCA. The data stream was divided into a series of non-overlapping ‘chunks’. Each label on the *x*-axis includes the top 10 features selected with the mRMR algorithm for a specific data chunk. The HATs are used as base classifiers. The figure reports the accuracy obtained within the chunk of instances (*InitAcc*); the accuracy obtained before updating the online models with instances derived from the TCGA cohort (*BeforeTCGA*); and the accuracy updated on the last unseen instance (*LastAcc*). The rightmost model is built when new, unseen instances arrive from TCGA

An important advantage offered by the FDD-ES approach is the possibility to analyze how the accuracy of the proposed online learning system change over time based on newly discovered genes (or biomarkers). Figure 4 shows the features that are selected when using the FDD-ES approach with a window of 500 instances and HATs as base classifiers. It should be noted that the reported genes correspond to the top 10 features selected within each chunk of data by the mRMR algorithm. It is interesting to observe how the accuracy of online models trained with newly discovered features can change over time. Indeed, some trained models will exhibit a decrease in accuracy, while other models will be able to improve their accuracy after acquiring new data. On the other hand, the proposed approach is based on the idea of using only the best *k* models for the prediction step, which will prevent the ES from the use of obsolete models. However, when patient profiles are derived from the TCGA-BRCA cohort, the accuracy of the online learning models start to decrease.

The system will improve again its accuracy after learning new informative feature sets. Remarkably, some features are selected across different chunks of data and datasets (e.g. between SCAN-B and TCGA). This suggests that the proposed FDD-ES can help achieve a better consensus on those biomarkers that are truly informative. Furthermore, it was observed that some features are more frequently selected, such as BUB1, or features that are preferred across different cohorts, such as AGR3. BUB1 expression is correlated with a poor clinical prognosis in patients with breast cancer and, in general, it has the potential to improve the prediction of breast cancer prognosis (Han *et al.*, 2015; Wang *et al.*, 2015). AGR3 is also associated with breast cancer progression (de Moraes *et al.*, 2022). It has been demonstrated that AGR3 could be used for early breast cancer detection from blood (Garczyk *et al.*, 2015). A univariate Cox proportional hazard regression model was used to assess the association of the union of biomarkers reported in Figure 4 with the overall survival of breast patients in the SCAN-B cohort.

**Fig. 5.**
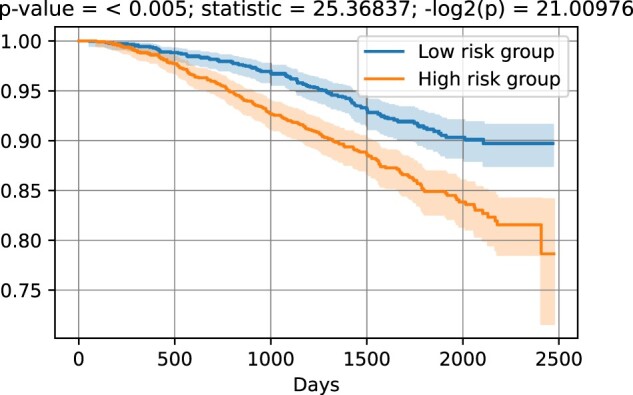
Kaplan–Meier analysis of the overall survival based on a prognostic score that distinguish low-risk from high-risk patients. The prognostic score is based on 16 genes, which were selected with univariate Cox analysis assessing the relationship between the biomarkers reported in Figure 4 and the survival cancer patients

This is an important step toward the evaluation of the clinical utility of new biomarker models. In total, 16 genes with the absolute value of the estimated coefficient >0.2 and *P*-value <0.01 were selected to build a risk score (for more details, see Section 2). This risk score was subsequently applied to classify patients into high- and low-risk groups, based on the median risk score (He and Zuo, 2019). Finally, the associations between the identified prognostic signatures and the overall survival, was evaluate by using the Kaplan–Meier analysis (see Fig. 5). The results show that the differences in survival time of low- and high-risk breast cancer patients are statistically significant, and that the high-risk group had a shorter overall survival than those in the low-risk group.

## 4 Conclusions

This study opens a new avenue of research and practical applications in omics-driven biomarker discovery. We strongly believe that a CL process is the ideal choice for ML-driven biomarker discovery. New patient data (e.g. RNA-seq based measures) and the results of previous prediction tasks (actual diagnoses) could be presented to the model, which would then transfer its previous knowledge to the new data, fine-tune its current task or even incrementally learn new learning tasks (e.g. new disease subtypes). Although online learning sounds ideal for medical reasons, in practice, many technical challenges exist. Here, we present a new computational framework that can be used to continuously evaluate and improve biomarker panels until they become mature enough to be included in the costly validations. As far as we know, this is the very first attempt to use online learning for biomarker discovery. It combines the previously published LFDD method with mRMR, which is a minimal-optimal feature selection algorithm. Our results show that certain biomarkers are informative across different chunks of data and patient cohorts. Most importantly, novel and more informative biomarkers predictive of breast cancer subtype can be discovered. Besides, our results suggest that current gold-standard panels include too many features, and that an online learning approach could help identify shorter lists of biomarkers, which in turn could ensure cost effectiveness and clinical translatability.

### Contributions

F.V. conceived and implemented the algorithm, interpreted the results and wrote the article. L.C. performed the Cox proportional hazards regression analysis, interpreted the results and wrote the article. The final manuscript was approved by all the authors.

## Funding

This study was supported by the Academy of Finland [grant agreements 336275, 332510]; and the Jane and Aatos Erkko Foundation.

*Conflict of Interest*: none declared.

## Supplementary Material

vbac074_Supplementary_Data

## Data Availability

The data underlying this article were retrieved from public repositories.
