# Supplementary material for: Identifying gene expression-based biomarkers in online learning environments
Source: Bioinform Adv. 2022 Oct 13;2(1):vbac074. doi: 10.1093/bioadv/vbac074 (PMC9710669; doi:10.1093/bioadv/vbac074)
Supplement: vbac074_Supplementary_Data [file vbac074_supplementary_data.docx]

Supplementary Figures

Identifying gene expression-based biomarkers in online learning environments

Luca Cattelani^1^ and Vittorio Fortino^1,*^

^1^Institute of Biomedicine, School of Medicine, University of Eastern Finland, Finland

**Supplementary Fig. 1 - Classification performances obtained by starting FDD-ES with two basic biomarker models and an empty biomarker set**.. The y-axis reports the balanced accuracy computed as new instances are received, varying the size of a data chunk (200/500) and the number of base models used for prediction (from one to three models). The two numbers above the graphs are respectively the size of chunks and the number of best models. The accuracies of the FDD models are in solid lines while the lines for their non-ensemble counterparts are in the same color but dotted. The dashed line on the online instances indicates the switch to SCAN-B data.

**Supplementary Fig. 2 - Classification performances obtained by starting FDD-ES with four basic biomarker models.** The y-axis reports the balanced accuracy computed as new instances are received, varying the size of a data chunk (200/500) and the number of base models used for prediction (from one to three models). The two numbers above the graphs are respectively the size of chunks and the number of best models. The accuracies of the FDD models are in solid lines while the lines for their non-ensemble counterparts are in the same color but dotted. The dashed line on the online instances indicates the switch to SCAN-B data.
